# Cohort vs case–control design for transformer-based prediction of asthma exacerbations in mild asthma

**DOI:** 10.1038/s41746-026-02624-3

**Published:** 2026-04-21

**Authors:** Fagen Xie, Eric J. Puttock, Matthew T. Slaughter, Robert S. Zeiger, William Crawford, Michael Schatz, Stanley Xu, Wansu Chen

**Affiliations:** 1 Department of Research and Evaluation, Kaiser Permanente Southern California, Pasadena, CA USA; 2 Kaiser Permanente Center for Health Research, Portland, OR USA; 3 Department of Allergy, Kaiser Permanente Southern California, San Diego, CA USA; 4 Department of Clinical Science, Kaiser Permanente Bernard J. Tyson School of Medicine, Pasadena, CA USA; 5 Department of Allergy, Kaiser Permanente Southern California, Harbor City, CA USA

**Keywords:** Computational biology and bioinformatics, Diseases, Health care, Medical research, Risk factors

## Abstract

Acute asthma exacerbation (AAE) is among the most serious outcomes of asthma, and accurate prediction of its risk remains a key challenge. Existing electronic health record-based prediction models have typically adopted either cohort or case–control sampling designs, yet their comparative performance has not been systematically evaluated. To address this gap, we developed transformer-based deep learning models to predict AAE among adults with mild asthma, directly comparing cohort and case–control designs using identical predictors and architecture across two large integrated healthcare systems, Kaiser Permanente Southern California (KPSC) and Kaiser Permanente Northwest (KPNW). Models were trained on retrospective data from KPSC and externally validated in KPNW. Mean area under the receiver operating characteristic curve (AUC) was 0.85 for case–control models and 0.70 (KPSC)/0.71 (KPNW) for cohort models. Both designs generalized well across systems, indicating robust feature learning and population transferability. Although calibration appeared well aligned within each analytical framework, absolute predicted probabilities diverged between designs, reflecting how event-enriched sampling inflates apparent risk and affects interpretability. These findings demonstrate that study design strongly influences model behavior and should be aligned with the intended use when developing predictive models for clinical deployment.

## Introduction

Asthma is a common chronic respiratory disease that affects people of all ages and can lead to significant health complications. One of the most serious outcomes, acute asthma exacerbation (AAE), can lead to hospitalization, long-term lung function decline, and death. Although exacerbations are often associated with moderate to severe asthma, up to 30–40% of asthma-related hospital visits and systemic corticosteroid use occur in patients classified as having mild asthma^1,2^, underscoring that exacerbation risk extends beyond severe disease. Early identification of patients with mild asthma who are at increased risk of future exacerbations is therefore critical for timely intervention and prevention.

Despite this burden, mild asthma remains underrepresented in both clinical and informatics research, and few predictive tools have been designed specifically for this population. Our previous work defined a clinically meaningful mild-asthma cohort within an integrated health-system setting and identified demographic and clinical correlates of AAE^3^, but it focused on risk-factor identification rather than predictive modeling. Whether future exacerbations can be predicted from routinely collected electronic health record (EHR) data in this large, low-risk population remains unknown.

Over the past several years, a growing number of machine-learning and deep-learning models have been developed to predict asthma exacerbations EHR data^4–15^. A recent systematic review identified 11 studies encompassing 23 prediction models for asthma exacerbation risk^16^, most of which were developed using cohort-based designs. While analytic approaches varied widely, few studies have explicitly compared cohort and case–control frameworks within the same population using identical predictors and modeling approaches. Because sampling design can influence outcome prevalence (event balance), temporal feature context, and calibration, understanding how cohort and case–control frameworks affect predictive performance is essential for reliable clinical application.

Although cohort and case–control sampling are often treated as interchangeable strategies for predictive modeling, they encode different inferential targets. Cohort sampling preserves outcome prevalence and the natural temporal ordering from a reference date, supporting absolute risk estimation in the source population, whereas case–control sampling enriches outcome events and anchors predictors near event times, often improving apparent discrimination but altering the meaning and transportability of predicted probabilities. For this reason, comparing these designs is essential for interpreting model performance and aligning prediction outputs with real-world clinical use.

To address these gaps, we developed transformer-based deep-learning models to predict AAE in adults with mild asthma and directly compared cohort and case–control designs using identical predictors and architecture. Leveraging harmonized EHR data from Kaiser Permanente Southern California (KPSC) and Kaiser Permanente Northwest (KPNW), we evaluated internal and external model performance, calibration, feature importance, and threshold-based trade-offs. This work provides a head-to-head evaluation of design choice for predictive modeling in mild asthma and clarifies its implications for model deployment and clinical translation.

## Results

### Study population

A total of 199,010 patients at KPSC and 33,441 at KPNW met eligibility criteria for mild asthma and were included in the cohort dataset (Supplementary Fig. 1). Within 1 year after the reference date, 6.5% of KPSC and 6.7% of KPNW patients experienced AAE.

We also constructed a case–control dataset that included 86,424 AAE cases and 309,517 non-AAE controls at KPSC, and 10,971 AAE cases and 60,120 controls at KPNW (Supplementary Fig. 2). Each case represented an ED or outpatient visit with a diagnostic code for asthma followed by an AAE within one year, whereas each control represented a visit with a diagnostic code for asthma without AAE during the following year.

### Baseline characteristics

Baseline demographic, clinical, and behavioral characteristics of patients in the cohort datasets at KPSC and KPNW are summarized in Table 1. Overall, patient profiles were broadly comparable across the two health systems, though notable differences were observed. Patients at KPSC were generally younger and more likely to identify as Hispanic, Black, or Asian/Pacific Islander, whereas those at KPNW were more likely to be White, current smokers, and less likely to have undergone aeroallergen testing or received influenza vaccination. At KPSC, 64.0% were women, 38.3% were Hispanic, 36.0% were White, 12.5% were Black, and 10.0% were Asian/Pacific Islander; the mean (SD) age was 43.6 (17.8) years and mean body-mass index (BMI) 30.8 (7.5). Among these patients, 5.3% were current smokers, 6.4% had undergone aeroallergen testing, and 72.0% had received influenza vaccination. At KPNW, 65.0% were women, 78.3% were White, 7.8% were Hispanic, 4.3% were Asian/Pacific Islander, and 3.3% were Black; the mean (SD) age was 46.5 (17.1) years and mean BMI 32.0 (8.2); 8.9% were current smokers, 2.1% had undergone aeroallergen testing, and 66.6% had received influenza vaccination.

**Table. 1:** Overall characteristics of the KPSC and KPNW cohort design datasets

Patient characteristics		KPSC			KPNW		
		AAE (%) *n* = 12,913	Non-AAE (%) *n* = 186,097	Overall (%) *n* = 199,010	AAE (%) *n* = 2235	Non-AAE (%) *N* = 31,176	Overall (%) *n* = 33,411
**Demographics**							
Age, in years, mean (SD)		46.1 (17.3)	43.5 (17.8)	43.6 (17.8)	49.3 (16.0)	46.3 (17.2)	46.5 (17.1)
Sex							
Female		69.8	63.6	64.0	72.8	64.4	65.0
Male		30.2	36.4	36.0	27.2	35.6	35.0
Race/ethnicity							
Non-Hispanic white		33.6	36.8	36.6	77.3	78.4	78.3
Non-Hispanic black		13.7	12.4	12.4	4.1	3.2	3.3
Hispanic		41.1	38.1	38.3	8.1	7.8	7.8
Non-Hispanic Asian/Pacific Islander		9.3	10.0	10.0	4.3	4.3	4.3
Others/multiple/unknown		2.3	2.7	2.7	6.2	6.3	6.3
Smoking							
Yes		5.7	5.2	5.3	10.5	8.8	8.9
Quit		21.4	20.2	20.2	26.9	26.7	26.7
Never		72.7	74.4	74.3	62.6	64.5	64.4
Unknown		0.2	0.2	0.2	0.0	0.0	0.0
Body mass index	Mean (SD)	32.5 (8.0)	30.8 (7.5)	30.9 (7.6)	33.8 (8.7)	31.9 (8.1)	32.0 (8.2)
	Median (IQR)	31.3 (26.8, 37.0)	29.7 (25.4, 35.0)	29.8 (25.5, 35.1)	32.4 (27.3, 39.0)	30.5 (25.9, 36.4)	30.7 (26.0, 36.6)
**Health behaviors/vitals/labs**							
Minutes exercised/week	Mean (SD)	187.6 (159.8)	198.7 (169.7)	197.9 (169.1)	97.0 (154.4)	114.0 (169.0)	112.5 (167.9)
	Median (IQR)	150 (90, 240)	150 (90, 240)	150 (90, 240)	40 (0, 150)	40 (0, 180)	40 (0, 180)
Eosinophil counts, cells/mcL	Mean (SD)	248.3 (325.3)	213.1 (281.5)	216.9 (276.0)	438.5 (1520.6)	370.4 (1520.9)	376.4 (1521.0)
	Median (IQR)	200 (100, 300)	170 (100, 280)	170 (100, 288)	190 (110, 310)	170 (100, 280)	170 (100, 280)
Aeroallergen test		8.0	6.3	6.4	2.9	2.0	2.1
Aeroallergen test - positive		4.5	3.7	3.7	NA	NA	NA
H pylori		2.2	2.0	2.0	0.5	0.5	0.5
Influenza		77.1	71.6	72.0	71.4	66.2	66.6
Asthma action plan		7.2	3.5	3.8	19.2	9.0	9.7
**Comorbidities (selected)**							
Pneumonia, influenza, and other acute lower respiratory infections		19.7	15.2	15.5	19.8	15.4	15.7
Gastroesophageal reflux disease		22.1	17.3	17.6	26.9	20.0	20.5
Atopic dermatitis		2.6	2.4	2.4	2.9	2.4	2.4
Allergic rhinitis		28.9	22.9	23.3	33.1	29.2	29.4
Chronic rhinitis		10.5	8.2	8.3	5.9	4.6	4.7
Chronic sinusitis		25.7	18.2	18.7	10.0	7.6	7.7
Nasal polyps		1.0	0.5	0.6	1.1	0.6	0.7
Post-nasal drip (upper airway cough syndrome)		5.8	4.8	4.8	2.4	2.5	2.5
Sleep disorders		18.9	14.6	14.9	32.7	24.7	25.3
Anxiety		23.9	20.1	20.3	26.0	22.3	22.5
Depression		19.7	16.5	16.7	27.6	21.9	22.3
Dementia		0.4	0.3	0.3	0.3	0.3	0.3
Anemia		11.1	10.0	10.0	10.2	7.6	7.8
Hyperlipidemia		34.8	31.0	31.3	25.7	22.6	22.8
Heart disease		10.0	8.3	8.4	12.1	9.7	9.9
Hypertension		30.9	25.4	25.7	33.3	26.2	26.7
Diabetes		14.2	11.9	12.0	13.9	11.2	11.4
Cerebrovascular disease		1.8	1.5	1.5	1.6	1.5	1.5
**Medications (selected)**							
Antiviral agents		11.2	9.2	9.4	11.4	9.1	9.2
Antianxiety agents		22.2	17.5	17.8	25.7	19.4	19.9
Antidepressants		29.3	23.8	24.2	42.9	33.0	33.7
Antipsychotics		5.3	4.2	4.3	7.5	5.3	5.5
Hypnotics		6.7	5.8	5.8	6.4	4.7	4.9
Antibacterial/antimicrobial agents		76.7	66.2	66.9	74.5	61.1	62.0
Antianginal agents		4.0	2.8	2.9	3.4	2.4	2.5
Antifungal agents		10.3	8.2	8.4	10.9	8.4	8.6
Antacids		10.7	8.5	8.6	2.5	1.9	2.0
Antihypertensive agents^a^		24.7	20.6	20.9	26.6	21.5	21.8
Diuretics		14.4	11.1	11.3	16.4	12.1	12.4
Beta blockers		13.3	11.2	11.3	16.7	13.4	13.6
Calcium channel blockers		10.9	8.3	8.5	8.5	6.8	6.9
Statin		25.6	22.3	22.5	23.1	18.7	19.0
Anticoagulants		8.1	6.2	6.3	7.3	6.00	6.1
Antidiabetics		12.2	10.4	10.5	12.8	10.5	10.7
Insulin		4.8	4.2	4.2	5.0	4.3	4.4
Sulfonylureas		5.3	4.6	4.6	4.0	3.5	3.5
Metformin		9.6	8.1	8.2	10.7	8.3	8.5
Ulcer drugs		38.8	31.9	32.4	33.1	25.0	25.6
Antiarrhythmics		2.0	1.4	1.5	0.3	0.5	0.5
SCS		59.8	38.8	40.1	62.1	35.2	37.0
ICS		37.2	22.4	23.4	50.4	34.1	35.2
ICS/LABA		15.2	6.9	7.4	11.5	5.1	5.6
LABA		0.3	0.2	0.2	0.9	0.3	0.4
LAMA		0.6	0.2	0.3	0.8	0.2	0.3
SABA		75.7	56.4	57.6	83.4	73.5	74.1
SABA/SAMA		0.8	0.4	0.5	0.04	0.04	0.04
SAMA		19.0	7.9	8.6	2.0	0.7	0.8
leukotriene modifier		8.9	4.9	5.2	5.9	3.6	3.7
Glipizide		4.8	4.2	4.3	4.0	3.4	3.4
Glyburide		0.5	0.3	0.4	0.04	0.1	0.1
Glimepiride		0.2	0.1	0.1	0.0	0.1	0.1

*KPSC* Kaiser Permanente Southern California, *KPNW* Kaiser Permanente Northwest, *AAE* acute asthma exacerbation, *Non-AAE* no acute asthma exacerbation, *ICS* inhaled corticosteroids, *LABA* long-acting beta-agonist, *LAMA* long-acting muscarinic antagonist, *SABA* short-acting beta-agonist, *SAMA* short-acting muscarinic antagonist, *SCS* systemic corticosteroids, *NA* not available.

^a^Not included calcium channel blockers, diuretics, beta blockers.

Common comorbidities (prevalence > 10%) in the two years before or on the reference date are summarized for KPSC and KPNW as follows, respectively: hyperlipidemia (31.3% vs 22.8%), hypertension (25.4% vs 26.7%), allergic rhinitis (23.3% vs 29.2%), anxiety (20.3% vs 22.5%), chronic sinusitis (18.7% vs 7.7%), gastroesophageal reflux disease (17.6% vs 20.5%), depression (16.7% vs 22.3%), pneumonia, influenza, and other acute lower respiratory infections (15.5% vs 15.7%), sleep disorders (14.9% vs 25.3%), diabetes (12.0% vs 11.4%), and anemia (10.0% vs 7.8%).

Frequently dispensed medications (prevalence > 10%) within two years before or on the reference date were likewise reported for KPSC and KPNW, respectively: short-acting beta-agonist (SABA, 57.6% vs 74.1%), systemic corticosteroids (SCS, 40.1% vs 37.0%), inhaled corticosteroids (ICS, 23.4% vs 35.2%), antibacterial/antimicrobial agents (66.9% vs 62.0%), ulcer drugs (32.4% vs 25.6%), antidepressants (23.8% vs 33.7%), statin (22.3% vs 19.0%), antihypertensive agents (20.6% vs 21.8%), antianxiety agents (17.8% vs19.9%), diuretics (11.1% vs 12.4%), beta blockers (11.2% vs 13.6%), and antidiabetics (10.4% vs 10.7%).

Across both health systems, patients who experienced an AAE tended to have higher prevalence of comorbidities, greater medication use, higher BMI, and lower physical activity compared with those without AAE.

Detailed distributions of demographics, comorbidities, medications, laboratory tests, vaccinations, exercise, and asthma action plan features in the case–control datasets are presented in Supplementary Table 1 for both KPSC and KPNW. Overall patterns were consistent with those observed in the cohort datasets (Table 1).

### Feature representation and temporal patterns

The overall distribution and structure of extracted feature tokens are summarized in Supplementary Table 2. As expected, the case–control datasets contained more feature tokens and visit-days per patient than the cohort datasets, reflecting the intentional enrichment of AAE cases. At KPSC, the mean (SD) number of tokens per patient was 94.1 in the cohort dataset and 107.7 in the case–control dataset, with a mean of 21.1 and 23.4 visit-days, respectively. Similar patterns were observed at KPNW, where case–control patients averaged 113.6 tokens and 24.8 visit-days per patient compared with 101.8 tokens and 22.5 visit-days in the cohort dataset.

Temporal patterns of feature prevalence and values are shown in Supplementary Figs. 3 and 4. In each figure, the x-axis represents time prior to the reference date, and the y-axis indicates either the proportion of binary features or the mean values of continuous features. In the cohort datasets, patients with AAE consistently demonstrated higher prevalence or mean values across nearly all features, except for exercise minutes per week, which showed an inverse relationship (Supplementary Fig. 3). In contrast, the case–control datasets revealed more variable and less monotonic trajectories: for several features, AAE cases had higher prevalence early in the look-back period but lower prevalence closer to the reference date (Supplementary Fig. 4). Features exhibiting this crossover pattern included aeroallergen testing, anemia, arrhythmias, anticoagulant and antipsychotic use, heart disease, and influenza vaccination.

### Feature importance

**Fig. 1:**
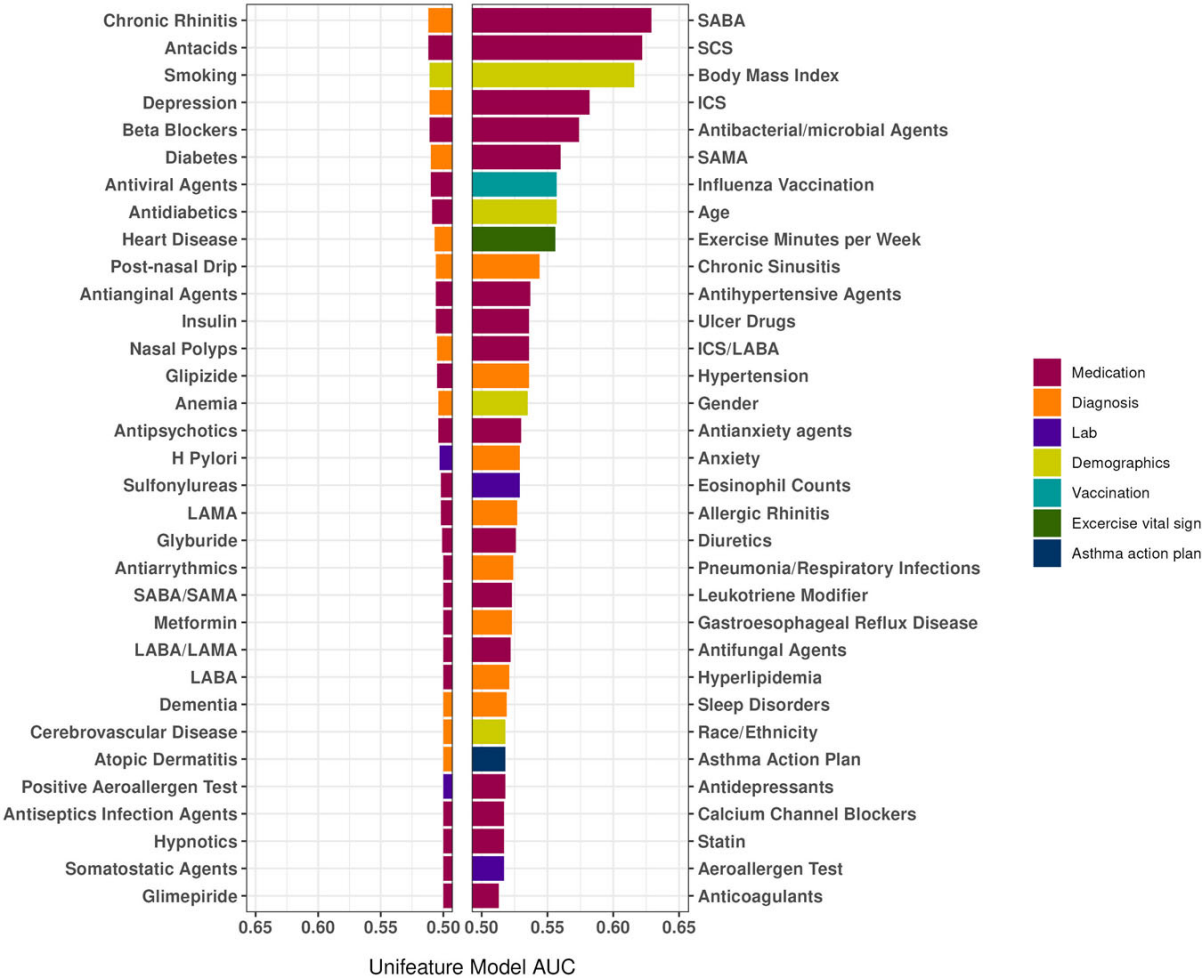
Feature importance based on unfeatured model AUCs for KPSC cohort dataset. Each bar shows the predictive performance (AUC) of a model using only the indicated feature. Bars are color-coded by feature domain. Top features included short-acting beta-agonist (SABA), systemic corticosteroids (SCS), body mass index, inhaled corticosteroids (ICS), and antibacterial/antimicrobial agents.

Feature importance rankings derived from single-feature models are presented in Fig. 1 (cohort) and Supplementary Fig. 5 (case–control). In the cohort dataset, the single-feature area under the receiver operating characteristic curve (AUC) ranged from 0.50 to 0.63, with the strongest predictors including SABA use, SCS use, BMI, inhaled corticosteroid (ICS) use, antibacterial or antimicrobial agents, short-acting muscarinic antagonist (SAMA) use, influenza vaccination, age, exercise minutes per week, and chronic sinusitis.

In the case–control dataset, AUCs ranged from 0.50 to 0.72. The most influential features largely overlapped with those in the cohort dataset, except that chronic sinusitis was replaced by inhaled corticosteroid/long-acting beta-agonist (ICS/LABA) combination therapy, and several predictors demonstrated modest shifts in relative ranking. Overall, medication-related variables and BMI consistently emerged among the highest-ranking contributors across both sampling designs.

### Model discrimination and calibration

**Fig. 2:**
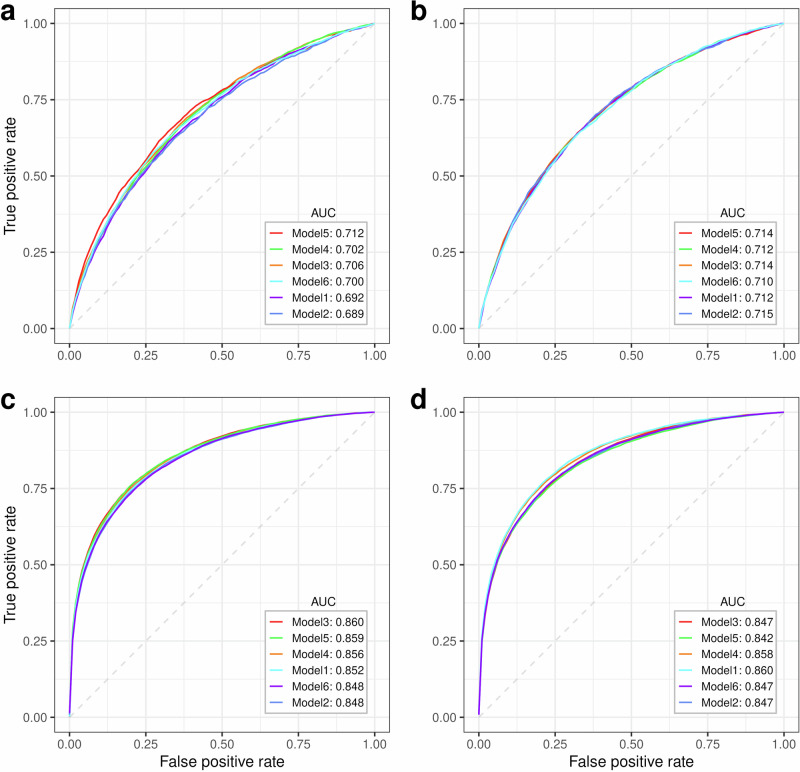
Discrimination performance of cohort-based and case–control-based models. Receiver operating characteristic (ROC) curves and area under the curve (AUC) values from six-fold cross-validation at KPSC and external validation at KPNW are summarized in the following panels: (**a**) cohort-based models at KPSC; (**b**) cohort-based models at KPNW; (**c**) case–control-based models at KPSC; (**d**) case–control-based models at KPNW. Case–control models showed higher apparent discrimination (AUC) than cohort models.

Model performance is summarized in Fig. 2 and Table 2. In the cohort models, AUCs from six cross-validation folds at KPSC ranged from 0.689 to 0.712 (mean ± SD 0.701 ± 0.009). External evaluation at KPNW yielded AUCs of 0.710–0.715 (mean ± SD 0.713 ± 0.002). In the case–control models, AUCs at KPSC ranged from 0.848 to 0.860 (mean ± SD 0.854 ± 0.005), and at KPNW from 0.842 to 0.860 (mean ± SD 0.850 ± 0.007).

**Table. 2:** Model performance (AUC and Brier score) in internal (KPSC) and external (KPNW) validation by study design

Model	KPSC (Internal Validation)		KPNW (External Validation)	
	AUC	Brier score	AUC	Brier score
**Cohort design**^a^				
Model 1	0.692	0.060	0.712	0.060
Model 2	0.689	0.050	0.715	0.060
Model 3	0.706	0.061	0.714	0.060
Model 4	0.702	0.065	0.712	0.060
Model 5	0.712	0.053	0.714	0.060
Model 6	0.700	0.055	0.710	0.060
Overall (mean, SD)	0.701 (0.009)	0.057 (0.006)	0.713 (0.002)	0.060 (0.000)
Model 7^b^	0.699	0.059	0.721	0.060
**Case-control design**^a^				
Model 1	0.852	0.111	0.860	0.088
Model 2	0.848	0.103	0.847	0.091
Model 3	0.860	0.115	0.847	0.091
Model 4	0.856	0.119	0.858	0.089
Model 5	0.859	0.107	0.842	0.091
Model 6	0.848	0.112	0.847	0.091
Overall (mean, SD)	0.854 (0.005)	0.111 (0.006)	0.850 (0.007)	0.090 (0.002)
Model 7^b^	0.856	0.111	0.858	0.088

*AUC* area under the receiver operating characteristic curve.

^a^Models 1–6 were developed using sixfold cross-validation within KPSC’s six service areas (each fold trained on 5 service areas and validated on the remaining 1). This produced six distinct models. Each of these six models was then independently applied to the full KPNW dataset, generating one external performance result per model.

^b^Model 7 was developed using a random 90%/10% train–validation split of the entire KPSC dataset (instead of cross-validation). The resulting single model was then applied once to the full KPNW dataset.

**Fig. 3:**
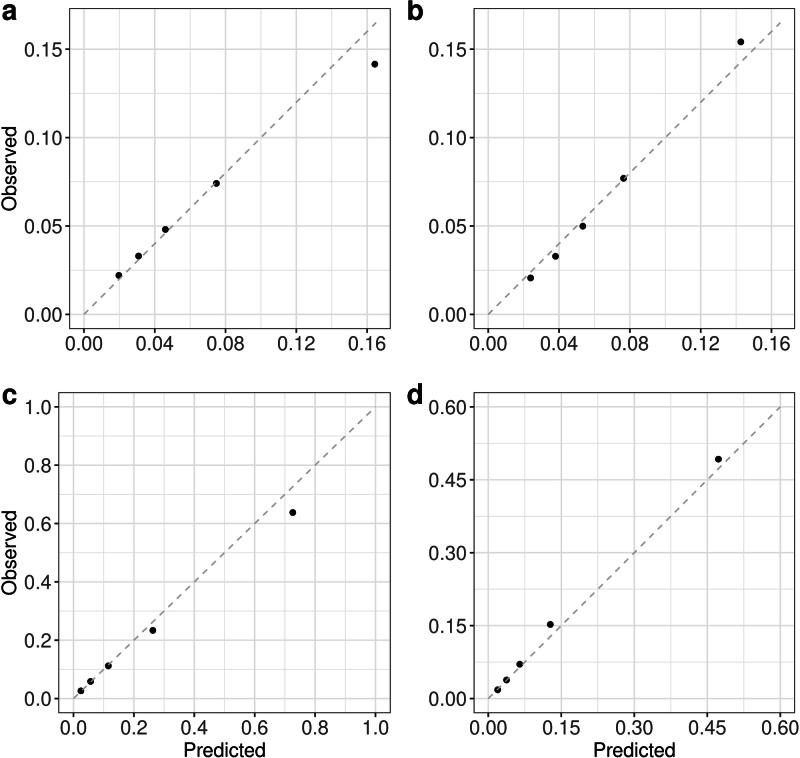
Calibration of cohort-based and case–control-based models. Calibration plots comparing observed versus predicted risk across quintiles of predicted probability are summarized in the following panels: (**a**) cohort-based models at KPSC; (**b**) cohort-based models at KPNW; (**c**) case–control-based models at KPSC; (**d**) case–control-based models at KPNW. Across the lower four quintiles, predicted and observed risks align closely at both sites. In the highest quintile (>80th percentile), KPSC models (cohort and case–control) slightly overestimated risk; at KPNW, both case-control and cohort models slightly underestimated.

Brier scores were lower for the cohort models (0.057 at KPSC; 0.060 at KPNW) than for the case–control models (0.111 at KPSC; 0.090 at KPNW). Calibration plots (Fig. 3) showed good alignment between predicted and observed risk across the lower four quintiles (≤80th percentile) for both designs and both sites. In the highest quintile (>80th percentile), predicted risks were slightly overestimated at KPSC for both designs, while at KPNW both case–control models and cohort models slightly underestimated observed risk.

Overall, both designs demonstrated consistent discrimination across sites, but the case–control models produced higher apparent AUCs and inflated absolute risk estimates, whereas the cohort models maintained better calibration relative to real-world event incidence.

### Threshold-based performance

Precision–recall–F1 trade-offs across prediction thresholds are shown in Supplementary Fig. 6. As thresholds increased, precision improved while recall declined, with F1 scores peaking at predicted-risk thresholds of 0.13 for cohort models and 0.37 for case–control models.

Table 3 summarizes operating characteristics at the top 1%, 5%, 10%, 15%, and 20% of ranked predicted risk. At the top 20% risk threshold, cohort models achieved sensitivity of 44.5% (KPSC) and 46.1% (KPNW) and positive predictive values (PPV) of 14.2% and 15.4%, respectively. Case–control models at the same threshold achieved sensitivities of 59.8% (KPSC) and 63.8% (KPNW) and PPVs of 63.8% and 49.2%, respectively.

**Table. 3:** Model performance at selected risk thresholds in internal (KPSC) and external (KPNW) validation datasets by study design

	KPSC (Internal Validation)					KPNW (External Validation)				
Threshold^a^	Top 20th	Top 15th	Top 10th	Top 5th	Top 1th	Top 20th	Top 15th	Top 10th	Top 5th	Top 1th
**Cohort design**										
Sensitivity (%)	44.5	37.0	28.0	16.8	4.6	46.1	38.5	28.8	16.7	4.3
Specificity (%)	81.7	86.5	91.2	95.8	99.2	81.9	86.7	91.4	95.8	99.2
PPV (%)	14.15	15.7	17.8	21.3	29.5	15.4	17.2	19.3	22.3	28.4
NPV (%)	96.6	95.3	94.9	94.4	93.9	95.5	95.2	94.7	94.1	93.5
Accuracy (%)	79.3	83.3	87.2	90.8	93.2	79.5	83.5	87.2	90.5	92.9
F1score	0.21	0.22	0.22	0.19	0.08	0.23	0.24	0.23	0.19	0.07
**Case–control design**										
Sensitivity (%)	59.8	50.5	38.1	21.3	4.6	63.8	55.6	44.0	26.4	6.2
Specificity (%)	90.8	94.6	97.6	99.4	100.0	88.0	92.4	96.2	98.9	100.0
PPV (%)	63.8	71.7	80.9	90.3	97.6	49.2	57.2	67.9	81.4	95.5
NPV (%)	89.2	87.5	85.3	82.3	79.4	93.0	91.9	90.4	88.0	85.4
Accuracy (%)	84.1	85.1	84.8	82.7	79.6	84.3	86.7	88.2	87.7	85.5
F1-score	0.62	0.60	0.52	0.34	0.09	0.56	0.56	0.53	0.40	0.12

*PPV* positive predictive value, *NPV* negative predictive value.

^a^Thresholds refer to the top-ranked percentiles of patients based on predicted risk (e.g., the top 20% with the highest predicted risk scores).

Across thresholds, sensitivity was consistently higher for case–control models, whereas PPV was more stable for cohort models relative to the background event rate.

## Discussion

This study developed transformer-based deep learning models to predict AAE in adults with mild asthma and directly compared models built using two study designs: cohort and case–control. Both designs used the same source population, predictors, and model architecture, and were evaluated through internal cross-validation at KPSC and external validation at KPNW. To our knowledge, this is the first risk-prediction study focused specifically on asthma exacerbations in a strictly defined mild-asthma population, offering new insights into how study design influences model behavior.

Our results show that case–control models achieved substantially higher apparent discrimination (AUCs greater than 0.80) than cohort models (AUCs around 0.70). When evaluated by quintiles of predicted risk, both designs showed close agreement between predicted and observed risks in the lower four quintiles and modest overestimation in the highest quintile. Case–control models produced much higher predicted probability values overall, reflecting their event-enriched sampling, whereas cohort-based models produced lower absolute probabilities that more closely matched real-world incidence rates. These findings demonstrate that design choice alone can meaningfully alter model behavior, which has critical implications for real-world deployment.

A central contribution of this work is the head-to-head evaluation of cohort and case–control frameworks for predictive modeling. Prior EHR-based studies of asthma exacerbation risk have varied widely in population characteristics, feature sets, and modeling methods. For example, Luo et al. developed a cohort-based prediction model within KPSC to predict asthma-related hospital encounters^6,7^, and Xiang et al. applied a time-sensitive attentive neural network to a cohort derived from the Cerner Health Facts database to forecast exacerbation^8^; but neither contrasted alternative sampling frameworks. A recent systematic review identified 11 studies encompassing 23 prediction models, but none performed a direct, within-population comparison of cohort-based versus event-enriched (case–control) sampling frameworks^16^. Our study, therefore, provides the first direct comparison of these two designs using identical predictors and architecture across two integrated health systems.

The observed performance differences likely stem from fundamental temporal distinctions between the two designs, which influence both feature representation and model learning. In the case–control datasets, all features are extracted from a fixed look-back window anchored to the event date for cases or a matched pseudo-event date for controls. Even controls who never experienced an exacerbation are artificially anchored to a pseudo-event, positioning their predictors immediately before an outcome. By aligning all patients, cases and controls alike, around a real or pseudo event, this design creates stronger apparent temporal signals. Such event-enriched alignment enables deep learning architectures such as Bidirectional Encoder Representations from Transformers (BERT) to learn sharp, event-linked feature associations, inflating discrimination but reducing generalizability. It also disrupts the natural temporal structure of the data, as reflected by the less monotonic feature trajectories observed. In contrast, the cohort design anchors patients at an eligible asthma visit (the reference date) and evaluates outcomes over a forward-looking prediction window, preserving the natural relationship between predictors and time-to-event. For patients who later experience an exacerbation, the event may occur months after the reference date; for those who never experience one, their predictors are never artificially tied to an outcome. This weaker temporal proximity better mirrors how prediction models would be applied in real-world settings and likely explains the observed differences in performance and feature dynamics between the two designs.

Although our calibration curves appeared well aligned within each analytic framework and site, this reflects design-specific rather than population-level calibration. If the case–control models were applied directly to general populations or in a real-world deployment setting without recalibration, divergence between predicted and observed risks would be expected, a phenomenon clearly demonstrated by Reps et al.^17^, whose outcome-enriched models markedly overestimated risk when evaluated against population data. This underscores a broader methodological principle: the alignment between a model’s sampling design and its evaluation setting fundamentally determines the interpretability of predicted probabilities, and calibration observed within one analytic framework should not be assumed to generalize across populations or study designs.

Another notable difference in performance was observed in Brier scores. Cohort models had substantially lower Brier scores than case–control models (0.057 vs 0.111 at KPSC; 0.060 vs 0.090 at KPNW). This difference likely reflects the much lower outcome prevalence and smaller predicted probabilities in the cohort models, which reduce squared prediction errors, rather than indicating inherently better calibration. Because the Brier score is sensitive to outcome prevalence, it is not directly comparable between these two designs and should not be interpreted as evidence of superior model accuracy. Rather, it is reported here for completeness.

Both designs demonstrated good generalizability between two large integrated healthcare systems, showing similar performance patterns across KPSC and KPNW. This supports the feasibility of building externally transportable risk models for mild asthma using routinely collected EHR data. Such cross-system validation is rarely performed in prior asthma prediction studies and strengthens the potential clinical utility of our models. The two designs also showed distinct threshold-based operating profiles. Case–control models consistently captured more true AAE cases among the highest risk predicted patients, whereas cohort models had lower sensitivity but more stable PPV relative to the background event rate. Cohort models therefore provided risk stratification more consistent with real-world incidence, which is crucial for identifying actionable thresholds for clinical interventions. From a design perspective, case–control models may be particularly useful for early-stage model development or feature discovery; however, their predicted probabilities typically require recalibration before being used for real-world risk stratification.

From a clinical perspective, the observed sensitivities and positive predictive values for both modeling approaches should be interpreted in the context of a low baseline risk of acute exacerbation in a strictly defined mild asthma population. Importantly, these performance characteristics do not negate the clinical relevance of the models, as discrimination and individual-level risk ranking remain strong. Rather than serving as diagnostic tools or replacing clinical assessment, the models are best viewed as clinical decision-support signals that can augment clinical awareness by highlighting patients with comparatively elevated risk. In practice, predicted risk estimates could be used to prompt clinicians to consider closer follow-up, review adherence and inhaler technique, or reinforce preventive strategies, while final decisions remain grounded in clinical judgment and patient-specific context. As with any clinical prediction tool, the balance between sensitivity and positive predictive value must be interpreted in light of clinical context, recognizing that predicted risk is intended to inform decision-making rather than dictate care.

Another important contribution of this study is the focus on risk prediction in the mild asthma population. Mild asthma has often been excluded or aggregated into general asthma cohorts, despite contributing substantially to the population burden of exacerbation. Many patients classified as having mild disease experience severe events, which represent a large absolute number of encounters in healthcare systems^18^. By restricting to a strictly defined mild asthma cohort, our study directly addresses this underexplored population. The performance of our models, particularly the good calibration and generalizability of the cohort-based model, shows that predictive modeling is feasible in this group and could support targeted preventive interventions.

Our findings provide practical guidance for epidemiologists and clinical prediction researchers when selecting sampling strategies for EHR-based risk modeling. Cohort-based designs are most appropriate when the goal is prospective risk estimation, calibration, and real-world deployment, as predicted probabilities more closely reflect population incidence. In contrast, event-enriched (case–control) designs may be useful during early-stage model development or comparative evaluation, particularly when discrimination and feature learning are prioritized, but absolute risk estimates from such models require careful interpretation or recalibration. These considerations highlight the importance of aligning study design, performance metrics, and interpretive goals when developing and evaluating clinical prediction models.

More broadly, the use of transformer-based models for clinical risk prediction raises considerations that are shared with other complex machine learning approaches in healthcare. These include challenges related to model interpretability, sensitivity to dataset shift across health systems, and the potential for differential performance across patient subgroups. While such issues are not unique to transformer architectures, they are particularly relevant for high-capacity models trained on longitudinal EHR data. When any machine learning model is considered for clinical deployment, appropriate governance, ongoing monitoring, and recalibration are essential to ensure reliable and equitable performance over time. These considerations reflect general challenges of applying advanced predictive models in healthcare rather than limitations specific to the present study or to transformer-based methods alone.

This study also has several limitations. Our models were trained and tested using routinely collected historical EHR data, and future external and real-world validation will be needed to assess their clinical impact. Our outcome definition relied on structured diagnosis codes and corticosteroid dispensing and may have missed mild events managed outside healthcare settings. Our models used only structured data, and incorporating unstructured clinical notes could further improve performance. Our feature importance analysis used univariate AUCs, which do not capture higher-order feature interactions learned by the transformer model. Although we tested generalizability across two large integrated systems, validation in other settings will be important to assess the transportability of our models. Finally, differences in temporal anchoring between the cohort and case–control designs reflect predictive modeling choices and should not be interpreted as causal effects.

In conclusion, this study developed deep learning models for predicting asthma exacerbations in a strictly defined mild asthma population and directly compared case–control and cohort study designs. Case–control models achieved higher apparent discrimination but produced inflated risk scores, whereas cohort models produced lower predicted probabilities more closely aligned with real-world incidence, making them more suitable for real-world clinical deployment. These findings demonstrate that study design exerts a strong influence on model behavior and should be aligned with the intended use when developing predictive models for clinical care.

## Methods

**Fig. 4:**
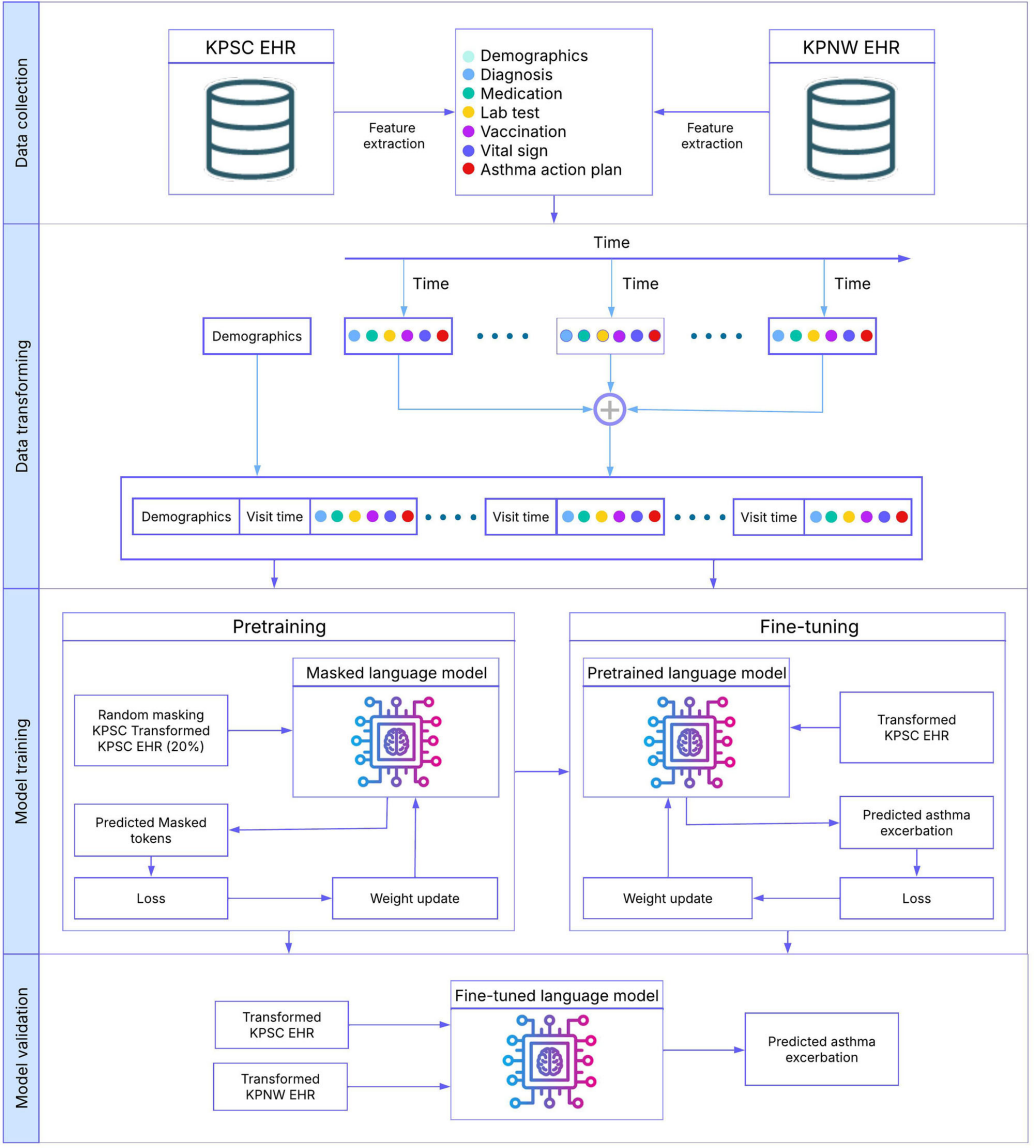
Overview of study workflow and transformer-based modeling framework. Structured EHR data from KPSC and KPNW were used to extract demographic, diagnosis, medication, lab, vaccination, vital sign, and asthma action plan features. Patient histories over 730 days were tokenized into temporal sequences. Models were pretrained using masked language modeling (MLM) objectives and then fine-tuned for asthma exacerbation prediction. External validation used KPNW data.

Figure 4 illustrates the study workflow and transformer-based modeling framework, including feature extraction and presentation, and model development and validation.

### Study design and setting

We developed and validated machine learning models to predict the risk of AAE using EHR of health plan enrollees at KPSC and KPNW. KPSC is a large integrated healthcare system that provides comprehensive care to more than 4.8 million enrollees across 15 medical centers and over 250 medical offices throughout Southern California. The KPSC membership is broadly representative of the general Southern California population^19^. Among these KPSC enrollees, 52% were Female, 48% were Male, 34% were non-Hispanic white, 43% Hispanic, 11% Asian/Pacific Islander, 9% African American, and 3% other or unknown. All aspects of care, including encounters (outpatient, inpatient, emergency department, virtual), laboratory results, medications, and claims for services received outside the system, are captured in the EHR. Model training and internal validation were conducted using KPSC data. Final models were externally evaluated using EHR data from KPNW, an organization which provides integrated care to approximately 600,000 enrollees in northwest Oregon and southwest Washington. Definitions of key study terms, including reference date and asthma exacerbation date, are provided in Supplementary Table 3.

The objective of this study was risk prediction rather than causal inference.

### Ethical approval

The study was conducted in strict compliance with the Health Insurance Portability and Accountability Act (HIPAA) and approved by the Institutional Review Boards of KPSC and KPNW with a waiver of signed informed consent (approval number 13414).

### Study datasets

We included adults aged 18–85 years who had a healthcare visit with an asthma diagnosis between 2013 and 2019. Eligible participants at KPSC and KPNW were used to construct two datasets based on cohort and case–control sampling designs. To ensure consistent temporal anchoring across designs, we use the term “reference date” throughout to denote either the cohort index date or the pseudo–reference date assigned in the case–control design.

The cohort dataset included all eligible patients who met previously described criteria for mild asthma, regardless of AAE status. Mild asthma was defined using a previously published algorithm based on asthma diagnosis codes and medication use patterns, as described in ref. ^3^. Briefly, eligible patients had documented asthma diagnoses and medication profiles consistent with mild disease severity, without evidence of persistent high-intensity controller therapy or markers of moderate-to-severe asthma during the baseline period. For patients with multiple health care visits with asthma diagnosis, one visit was randomly selected (reference date). This selection avoids correlated repeated measures and prevents systematic bias toward visits occurring earlier or later in the disease course. Sample sizes are shown in Supplementary Fig. 1. For all cohort participants, the reference date was defined as the date of the selected qualifying asthma visit.

The outcome was defined as the first AAE occurring within 365 days after the reference date, excluding any AAE events within the first 30 days to avoid misclassifying care related to the reference episode as a new exacerbation. An AAE was determined in two ways^3^: (1) a hospitalization, ED visit, or hospital-based observation with a principal discharge diagnosis of asthma or wheezing, or other specific respiratory conditions as the principal diagnosis, with either asthma exacerbation or status asthmaticus as the secondary diagnosis; or (2) receipt of oral, intramuscular, or intravenous corticosteroid therapy with asthma as the principal or primary visit diagnosis (or as the diagnosis linked to the systemic corticosteroid order).

The case–control dataset included all qualified mild asthma visits followed by an AAE within 365 days (cases) and a random sample of qualified mild asthma visits not followed by an AAE within 365 days (controls). Sample sizes are shown in Supplementary Fig. 2.

For controls, the pseudo-reference date was assigned equal to the AAE date of a selected case. This ensured that feature extraction used the same look-back duration for cases and controls, while the calendar time windows differed by construction. This design intentionally enriches AAE events relative to the cohort dataset.

### Feature extraction and representation

Candidate predictive features were selected based on literature review and expert input from allergy and asthma specialists. Feature domains included demographics, diagnoses, medications, laboratory tests, exercise vital sign, vaccinations, and asthma action plan (listed in Supplementary Table 4). For each patient, all features recorded within 730 days before or on the reference date were extracted from the EHR system at the day level and ordered chronologically to construct a temporal feature sequence^20^:

Patient feature sequence = {DT, TE, D1, TE, D2, TE, … Dn, TE}

where DT represents demographic tokens, TE is a special “end” token, and Di is the set of feature tokens recorded on day i ({ti, featurei1, featurei2, …}) (Fig. 4).

We ranked features by their univariate discriminative ability, calculated as the AUC from a single-feature model trained using only that feature.

Features were summarized overall and by AAE status for both study sites. Temporal trajectories over the 2-year feature collection window at KPSC were also plotted to compare patients with versus without AAE.

Missing data were handled implicitly within the modeling pipeline. No explicit imputation was performed. Continuous features with missing values were retained as missing, and the transformer architecture accommodated missingness through the absence of corresponding feature tokens during sequence construction. Binary features reflected the presence or absence of recorded events in the EHR.

### Training, validation, and external testing

We used transformer-based Bidirectional Encoder Representations from Transformers (BERT) architecture^21,22^ to develop models for both study designs. Each patient’s feature sequence was tokenized, embedded and fed into the model. Model development involved two stages: 1) feature pretraining using masked language modeling (MLM) on KPSC sequences, with 20% of tokens randomly masked for pretraining evaluation; and 2) downstream AAE prediction through supervised fine-tuning, optimized via six-fold cross-validation for both cohort and case–control datasets.

At KPSC, data were partitioned by six geographic medical service areas. For each design (cohort and case–control), we performed six-fold cross-validation by iteratively training on five service areas and validating on the held-out area. This yielded six fold-specific models per design, and internal performance was summarized across folds.

For external testing, each of the six fold-specific KPSC models was applied to the full KPNW dataset, and external performance was summarized across the six models.

As a sensitivity analysis, we also trained a single model per design using a random 90%/10% train–validation split of the full KPSC dataset and then applied that model once to the full KPNW dataset.

Model hyperparameters are listed in Supplementary Table 5.

### Performance evaluation

Model discrimination was assessed using AUC and Brier score. Calibration was evaluated using calibration plots constructed by quintiles of predicted risk (1–20th, 21–40th, 41–60th, 61–80th, and 81–100th percentiles).

We also calculated sensitivity, specificity, positive predictive value (PPV), negative predictive value (NPV), accuracy, and F1 score at the top 1%, 5%, 10%, 15%, and 20% of ranked predicted risk. PPV values were compared with the observed AAE prevalence to estimate fold enrichment across both internal and external validation datasets.

In addition, feature-level discrimination was quantified by calculating the AUC from single-feature models, each trained using only one predictor.

### Computational environment

All analyses were conducted using Python 3.10 (Python Software Foundation). Model training and KPSC internal evaluation were performed on a Lambda workstation equipped with 1 TB RAM, an AMD Threadripper Pro 3975WX 32-core 3.50 GHz CPUs, and four RTX A6000 GPUs (49 GB RAM each). KPNW external validation was conducted on a Windows server (16 cores at 3.0 GHz CPUs and 438 GB RAM).

All required packages were installed in accordance with the published BERT model repository^23^. Pretraining and fine-tuning were parallelized across four GPUs. Final trained models were securely transferred to KPNW for external validation.

## Supplementary information

PEARL risk prediction model development supplementary file R1

## Data Availability

Anonymized data that support the findings of this study may be made available from the investigative team under the following conditions: (1) agreement to collaborate with the study team on all publications, (2) provision of external funding for administrative and investigator time necessary for this collaboration, (3) demonstration that the external investigative team is qualified and has documented evidence of training for human subjects protections, and (4) agreement to abide by the terms outlined in data use agreements between institutions.

## Code availability

The deep learning code developed for this study is available at https://github.com/KPSCRE-Informatics/PEARL-BERT.
