# Supplementary material for: Cohort vs case–control design for transformer-based prediction of asthma exacerbations in mild asthma
Source: NPJ Digit Med. 2026 Apr 21;9:481. doi: 10.1038/s41746-026-02624-3 (PMC13287595; doi:10.1038/s41746-026-02624-3)

Supplementary Table 1. Overall characteristics of case-control design datasets at KPSC and KPNW.

Patient characteristics		KPSC			KPNW		
		AAE (%) n=86,424	Non-AAE (%) n=309,517	Overall (%) n=395,941	AAE (%) n=10,971	Non-AAE (%) n=60,120	Overall (%) n=71,091
**Demographics(n,%)**							
Age, in years, mean (SD)		48.6 (16.7)	42.7 (18.07)	44.0 (18.0)	50.1 (16.0)	45.4 (17.7)	46.1 (17.5)
Sex							
Female		72.4	61.6	63.9	73.8	63.9	65.4
Male		27.6	38.4	36.1	26.2	36.1	34.6
Race/ethnicity							
Non-Hispanic white		33.4	39.2	38.7	77.9	77.2	77.3
Non-Hispanic black		14.7	13.4	13.5	3.9	3.3	3.4
Hispanic		40.5	35.1	35.6	8.5	7.5	7.6
Non-Hispanic Asian/Pacific Islander		9.3	9.6	9.5	3.6	4.0	3.9
Others/multiple/unknown		2.1	2.7	2.7	6.2	8.0	7.7
Smoking							
Yes		5.2	6.4	6.2	10.8	11.2	11.1
Quit		23.0	20.6	21.1	29.8	27.2	27.6
Never		71.7	72.6	72.4	59. 4	61.6	61.3
Unknown		0.1	0.4	0.4	0.0	0.0	0.0
Body mass index	mean (SD)	33.0 (8.1)	30.7 (7.7)	31.3 (7.9)	34.7 (9.0)	31.4 (8.2)	32.0 (8.5)
	Median (IQR)	31.8 (27.2, 37.5)	29.4 (25.1, 34.8)	30.1 (25.6, 35.6)	33.3 (28.1, 40.0)	30.0 (25.40, 35.74)	30.6 (25.9, 36.7)
**Health behaviors/vitals/labs**							
Minutes exercised/week	mean (SD)	182.4 (155.7)	197.4 (168.9)	193.4 (165.6)	90.2 (153.6)	106.9 (165.5)	103.6 (163.4)
	Median (IQR)	150 (90, 240)	150 (90, 240)	150 (90, 240)	0 (0, 140)	0 (0, 160)	0 (0, 150)
Eosinophil counts, cells/mcL	mean (SD)	243.4 (284.5)	224.1 (832.4)	228.6 (742.3)	457.6 (1701.4)	530.9 (6139.0)	517.3 (5590.7)
	Median (IQR)	200 (100, 300)	160 (100, 270)	170 (100, 290)	190 (110, 300)	160 (90, 280)	170 (90, 290)
Aeroallergen test		7.3	6.5	6.7	3.6	2.6	2.7
Aeroallergen test - positive		4.1	3.8	3.9	NA	NA	NA
H pylori		2.5	1.9	2.1	0.6	0.6	0.6
Influenza		77.1	70.0	71.5	71.5	63.7	64.9
Asthma action plan		7.7	1.5	3.6	16.3	4.2	6.1
**Comorbidities (selected)**							
Pneumonia, influenza, and other acute lower respiratory infections		20.6	13.7	15.2	22.7	13.5	14.9

Gastroesophageal reflux disease	25.6	18.4	20.00	29.1	20.9	22.2
Atopic dermatitis	2.6	2.2	2.3	2.1	2.0	2.0
Allergic rhinitis	27.9	20.9	22.4	30.8	23.4	24.6
Chronic rhinitis	11.2	7.4	8.2	6.4	4.2	4.5
Chronic sinusitis	26.5	14.9	17.4	11.4	6.4	7.2
Nasal polyps	1.2	0.6	0.7	0.9	0.5	0.5
Post-nasal drip (upper airway cough syndrome)	5.8	4.3	4.6	2.9	2.0	2.2
Sleep disorders	21.3	15.5	16.7	34.5	24.7	26.2
Anxiety	25.7	21.7	22.6	29.1	25.1	25.7
Depression	22.2	17.4	18.5	30.6	24.0	25.0
Dementia	0.4	0.7	0.6	0.7	1.0	1.0
Anemia	12.6	12.4	12.5	11.7	10.4	10.6
Hyperlipidemia	40.3	31.6	33.5	28.6	22.4	23.3
Heart disease	11.7	10.2	10.6	14.8	12.5	12.9
Hypertension	36.2	26.5	28.6	35.6	26.5	27.9
Diabetes	17.2	13.5	14.3	17.6	12.5	13.3
Cerebrovascular disease	2.0	2.1	2.1	2.3	2.3	2.3
**Medications (selected)**						
Antiviral agents	13.2	10.1	10.8	12.5	9.1	9.6
Antianxiety agents	24.5	18.5	19.8	28.1	20.9	22.0
Antidepressants	32.7	25.1	26.7	45.1	34.4	36.1
Antipsychotics	5.9	5.5	5.6	9.4	7.4	7.7
Hypnotics	7.7	6.6	6.8	6.4	5.0	5.2
Antibacterial or antimicrobial agents	82.8	68.1	71.3	75.0	55.8	58.8
Antianginal agents	4.8	3.7	3.9	4.1	3.1	3.3
Antifungal agents	11.3	8.3	9.0	12.5	8.2	8.8
Antacids	12.1	12.4	12.3	3.6	3.5	3.5
Antihypertensive agents[¶]	28.9	22.2	23.6	29.5	21.9	23.1
Diuretics	17.1	12.1	13.2	18.9	13.1	14.0
Beta blockers	15.0	12.9	13.3	18.5	15.1	15.6
Calcium channel blockers	12.7	9.6	10.3	10.1	7.9	8.3
Statin	30.3	23.1	24.6	25.0	19.2	20.1
Anticoagulants	9.9	9.2	9.3	11.2	9.6	9.8
Antidiabetics	16.0	13.3	13.9	17.0	12.2	13.0
Insulin	5.8	5.5	5.6	8.2	6.4	6.7
Sulfonylureas	6.4	5.4	5.6	5.1	3.7	3.9

Metformin	11.5	8.9	9.5	13.4	8.8	9.5
Ulcer drugs	44.2	35.9	37.7	36.0	26.8	28.2
Antiarrhythmics	2.1	2.0	2.0	0.7	0.7	0.7
SCS	72.7	38.5	45.9	59.1	23.8	29.2
ICS	47.3	27.9	32.1	43.1	24.0	27.0
ICS/LABA	22.6	9.6	12.4	11.1	4.8	5.8
LABA	0.4	0.3	0.3	0.7	0.3	0.4
LAMA	0.9	0.4	0.5	1.6	2.0	2.0
SABA	87.7	72.2	75.6	80.6	64.5	67.0
SABA/SAMA	0.9	0.6	0.7	0.1	0.04	0.1
SAMA	27.7	8.8	27.7	2.5	1.3	1.5
Leukotriene modifier	12.3	7.5	8.6	4.5	3.0	3.2
Glipizide	6.0	5.0	5.2	5.0	3.6	3.8
Glyburide	0.4	0.4	0.4	0.1	0.1	0.1
Glimepiride	0.2	0.2	0.2	0.1	0.1	0.1

¶ not included calcium channel blockers, diuretics, beta blockers
KPSC: Kaiser Permanente Southern California
KPNW: Kaiser Permanente Northwest
AAE: acute asthma exacerbation
Non-AAE: no acute asthma exacerbation
ICS: inhaled corticosteroids
LABA: long-acting beta-agonist
LAMA: long-acting muscarinic antagonist
SABA: short-acting beta-agonist
SAMA: short-acting muscarinic antagonist
SCS: systemic corticosteroids
NA: not available

Supplementary Table 2. Summary of feature token and visit-day counts in the cohort and case–control datasets at KPSC and KPNW.

Metric	KPSC			KPNW		
	AE	Non-AE	All	AE	Non-AE	All
**Cohort design dataset**						
Total feature tokens	1,509,255	17,221,309	18,730,564	295,554	3,105,636	3,401,190
Feature token per patient (median, IQR)	86 (47, 151)	63 (33, 119)	65 (34, 121)	97 (53, 171)	68 (34, 130)	69 (35, 133)
Feature token per patient (mean, SD)	116.9 (110.0)	92.5 (94.1)	94.1 (95.4)	135.1 (119.4)	99.6 (101.4)	101.8 (103.3)
Total visit days with feature tokens	329,207	3,869,648	4,198,855	64,673	687,301	751,974
Visit days with feature tokens per patient (median, IQR)	19 (10, 34)	14 (7, 28)	15 (7, 28)	22 (12, 38)	15 (7, 30)	16 (8, 30)
Visit days with feature tokens per patient (mean, SD)	25.5 (23.1)	20.8 (20.8)	21.1 (20.9)	28.9 (25.1)	22.0 (21.5)	22.5 (21.8)
**Case-control design dataset**						
Total feature tokens	11,444,588	31,187,158	42,631,746	1,628,249	6,445,862	8,074,111
Feature token per patient (median, IQR)	100 (57, 170)	64 (33, 123)	71 (37, 135)	107 (58, 189)	65 (31, 134)	71 (33, 145)
Feature token per patient (mean, SD)	132.4 (117.4)	100.8 (121.5)	107.7 (121.4)	148.4 (141.6)	107.2 (126.6)	113.6 (129.9)
Total visit days with feature tokens	2,457,968	6,808,548	9,266,516	356,751	1,405,437	1,762,218
Visit days with feature tokens per patient (median, IQR)	22 (12, 38)	14 (7, 28)	16 (8, 31)	25 (13, 42)	15 (7, 30)	16 (7, 33)
Visit days with feature tokens per patient (mean, SD)	28.4 (24.8)	22.0 (25.2)	23.4 (25.3)	32.0 (28.8)	23.4 (25.7)	24.8 (26.4)

Notes: A feature token was defined as any indicator of diagnosis, medication, laboratory test, demographics, exercise vital sign, vaccination, and asthma action plan.

KPSC: Kaiser Permanente Southern California

KPNW: Kaiser Permanente Northwest

AAE: acute asthma exacerbation

Non-AAE: no acute asthma exacerbation

SD: standard deviation

IQR: interquartile Range

Supplementary Table 3. Terms used for acute asthma exacerbation prediction.

Term	Description
Reference date	The date of the selected visit with an asthma diagnosis within the study period
Feature collection window	730 days prior to/on the index date; data from this window were used for prediction
Asthma exacerbation time window	365 days after the index date
Asthma exacerbation date	The first date of an acute asthma exacerbation within the exacerbation time window
Case group	Patients with mild asthma who had an acute asthma exacerbation within the exacerbation time window
Control group	Patients with mild asthma who did not have an acute asthma exacerbation within the exacerbation time window
Patient feature sequence	A chronological sequence of feature tokens collected within 730 days before the index date

Supplementary Table 4. Candidate predictor features extracted during the 730 days feature collection window prior to the index date

Feature category	Feature name
Demographics	Sex
Demographics	Race/ethnicity
Demographics	Age
Demographics	Smoking status
Demographics	Body mass index
Diagnoses	Allergic rhinitis
Diagnoses	Anemia
Diagnoses	Anxiety
Diagnoses	Anxiety - disorders
Diagnoses	Anxiety - obsessive-compulsive disorder
Diagnoses	Anxiety - phobic disorders
Diagnoses	Asthma
Diagnoses	Atopic dermatitis
Diagnoses	Cerebrovascular disease
Diagnoses	Chronic rhinitis
Diagnoses	Chronic sinusitis
Diagnoses	Dementia
Diagnoses	Depression
Diagnoses	Diabetes
Diagnoses	Gastroesophageal reflux disease
Diagnoses	Heart disease
Diagnoses	Hyperlipidemia
Diagnoses	Hypertension
Diagnoses	Nasal polyp
Diagnoses	Pneumonia/respiratory infections
Diagnoses	Post nasal drip
Diagnoses	Sleep disorders
Diagnoses	Sleep disorders, insomnia
Diagnoses	Sleep disorders, others
Diagnoses	Sleep disorder, sleep apnea
Medications	Antibacterial or antimicrobial agents
Medications	Antacids
Medications	Antianginal agents
Medications	Antianxiety agents
Medications	Antiarrhythmics
Medications	Anticoagulants
Medications	Antidepressants
Medications	Antidiabetics
Medications	Antifungal agents
Medications	Antihyperlipidemic agents

Medications	Antihypertensive agents
Medications	Anti-obesity agents
Medications	Antipsychotics
Medications	Antiseptics disinfectants
Medications	Antiviral agents
Medications	Beta blockers
Medications	Calcium channel blockers
Medications	Diuretics
Medications	Glimepiride
Medications	Glipizide
Medications	Glyburide
Medications	Hypnotics
Medications	Inhaled corticosteroids
Medications	Inhaled corticosteroids/long-acting beta-agonist
Medications	Insulin
Medications	long-acting beta-agonist
Medications	Long-acting muscarinic antagonist
Medications	Metformin
Medications	Short-acting beta-agonist
Medications	Short-acting beta-agonist/Short-acting muscarinic antagonist
Medications	Short-acting muscarinic antagonist
Medications	Systemic corticosteroids
Medications	Somatostatic agents
Medications	Sulfonylureas
Medications	Ulcer drugs
Medications	Asthma agents
Medications	Biologic
Medications	Leukotriene
Medications	Theophylline
laboratory tests	Eosinophil count
laboratory tests	Aeroallergen test
laboratory tests	Positive aeroallergen test
laboratory tests	H Pylori
Vaccination	Influenza
Exercises	Exercise minutes per week
Asthma action plan	Asthma action plan
Visit date	Visit date

Supplementary Table 5. Hyperparameters used for pretraining and asthma exacerbation prediction model development.

Task	Vocabulary size	Masking rate (% tokens masked)	Sequence length	Learning rate	Mini-batch size	Training epochs
Masked Language model (pretraining)	2417	10%	128, 256, 512	Model auto-tunning	8, 16, 32, 64	10, 20, 40
Acute asthma exacerbation classification (fine-tuning)	2417	NA	128,256, 512	1e-5, 5e-6, 2e-6	32, 64	2, 3,4,5

Supplementary Figure 1. Study cohort construction for cohort design dataset**.** Consort diagram showing inclusion/exclusion flow from initial asthma-related visits to the final mild asthma cohort at KPSC (n = 199,010) and KPNW (n = 33,411).

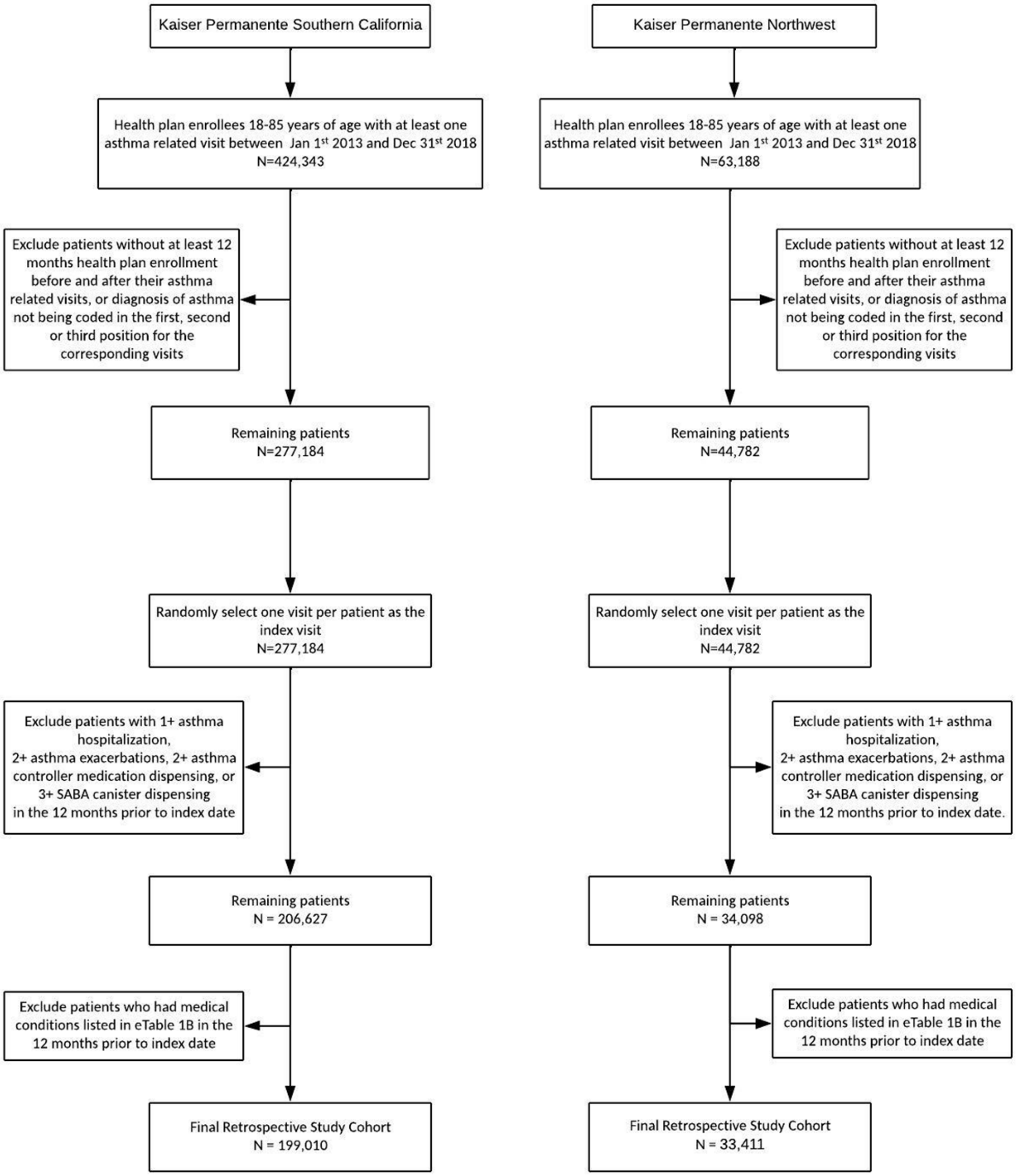

Supplementary Figure 2. Study cohort construction for case–control design dataset**.** Consort diagram showing selection of acute asthma exacerbation (AAE) cases and non-AAE controls, including assignment of reference dates for controls, at KPSC and KPNW.

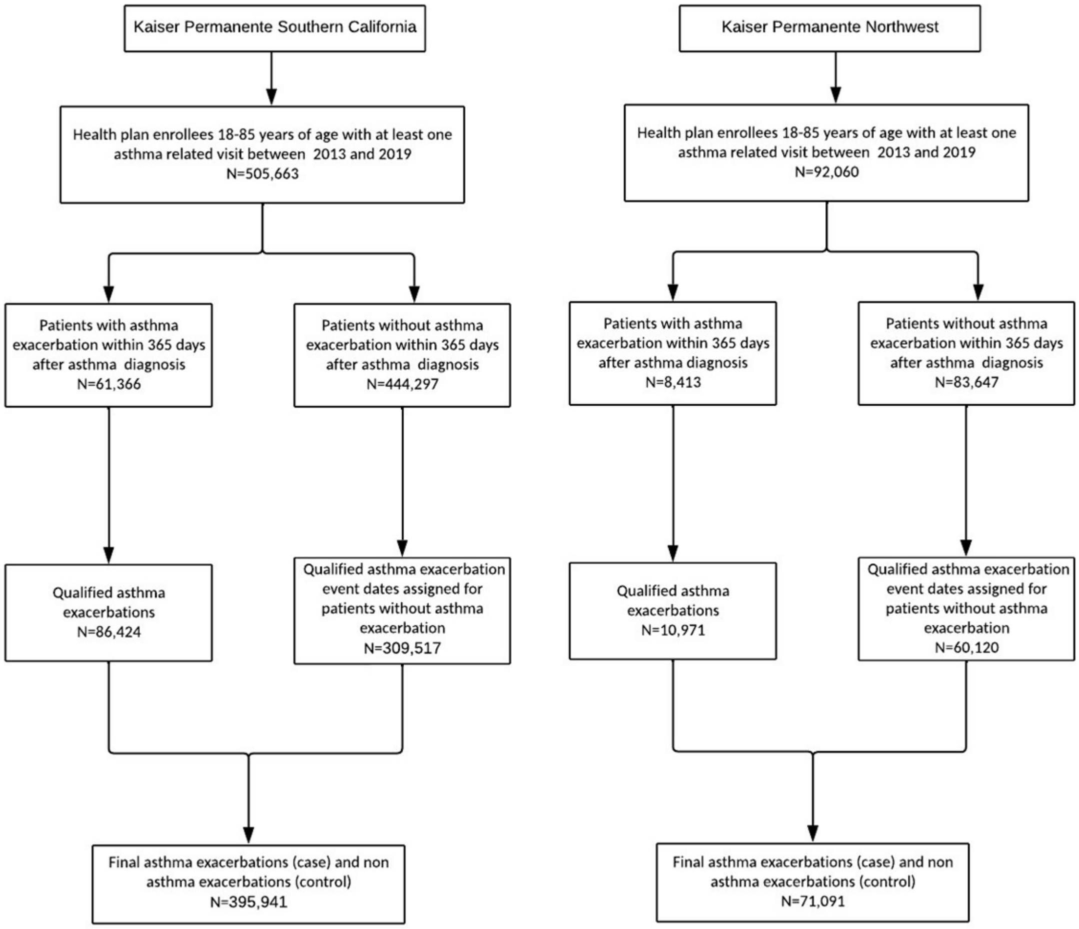

Supplementary Figure 3. Feature trajectories in the KPSC cohort dataset. Temporal trajectories of feature prevalence or mean values over the 24 months before reference date in the KPSC cohort dataset, stratified by AAE (red) and non-AE (purple).

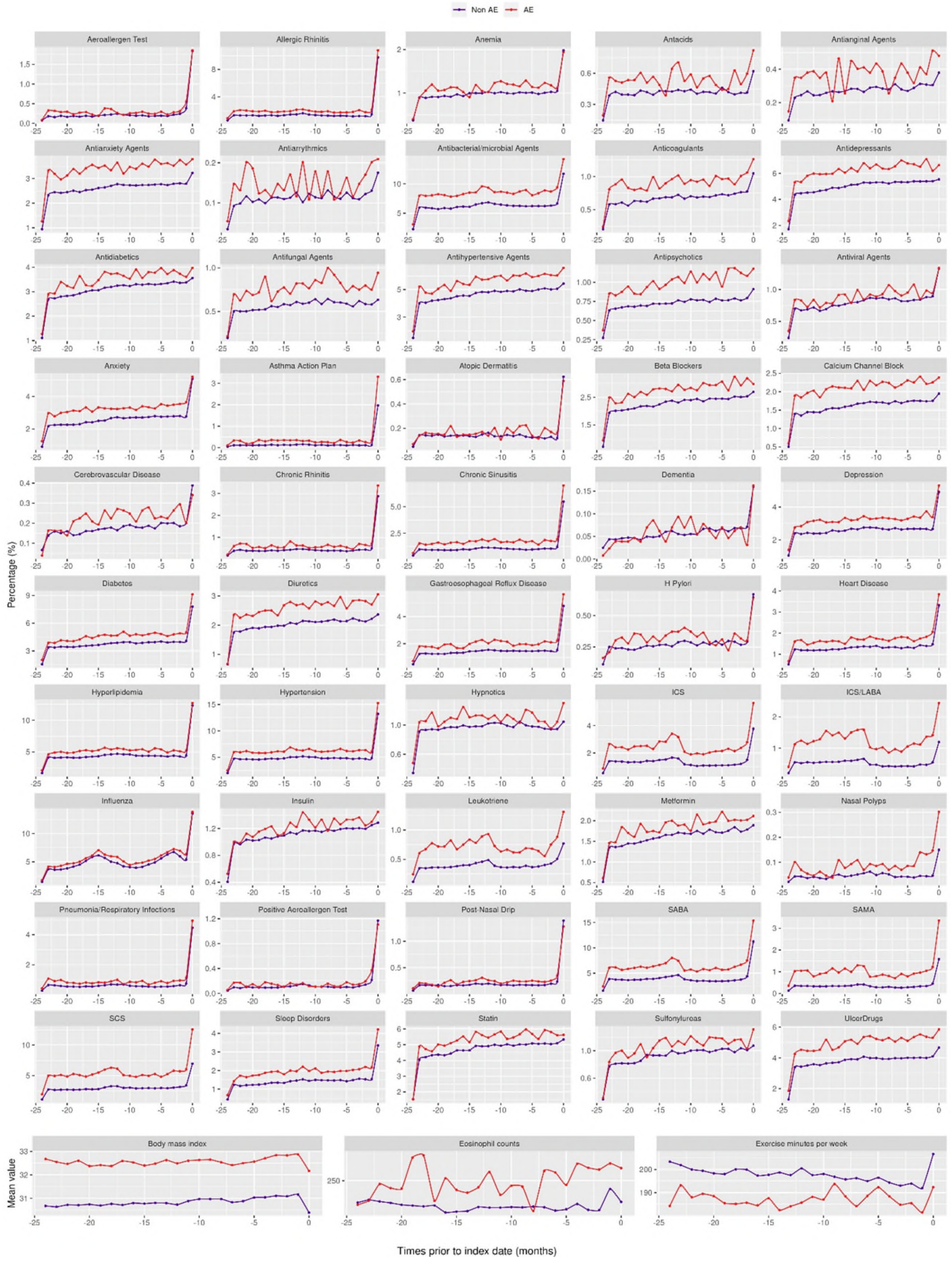

Supplementary Figure 4. Feature trajectories in the KPSC case–control dataset. Temporal trajectories of feature prevalence or mean values over the 24 months before reference date in the KPSC case–control dataset, stratified by AAE (red) and non-AAE (purple).

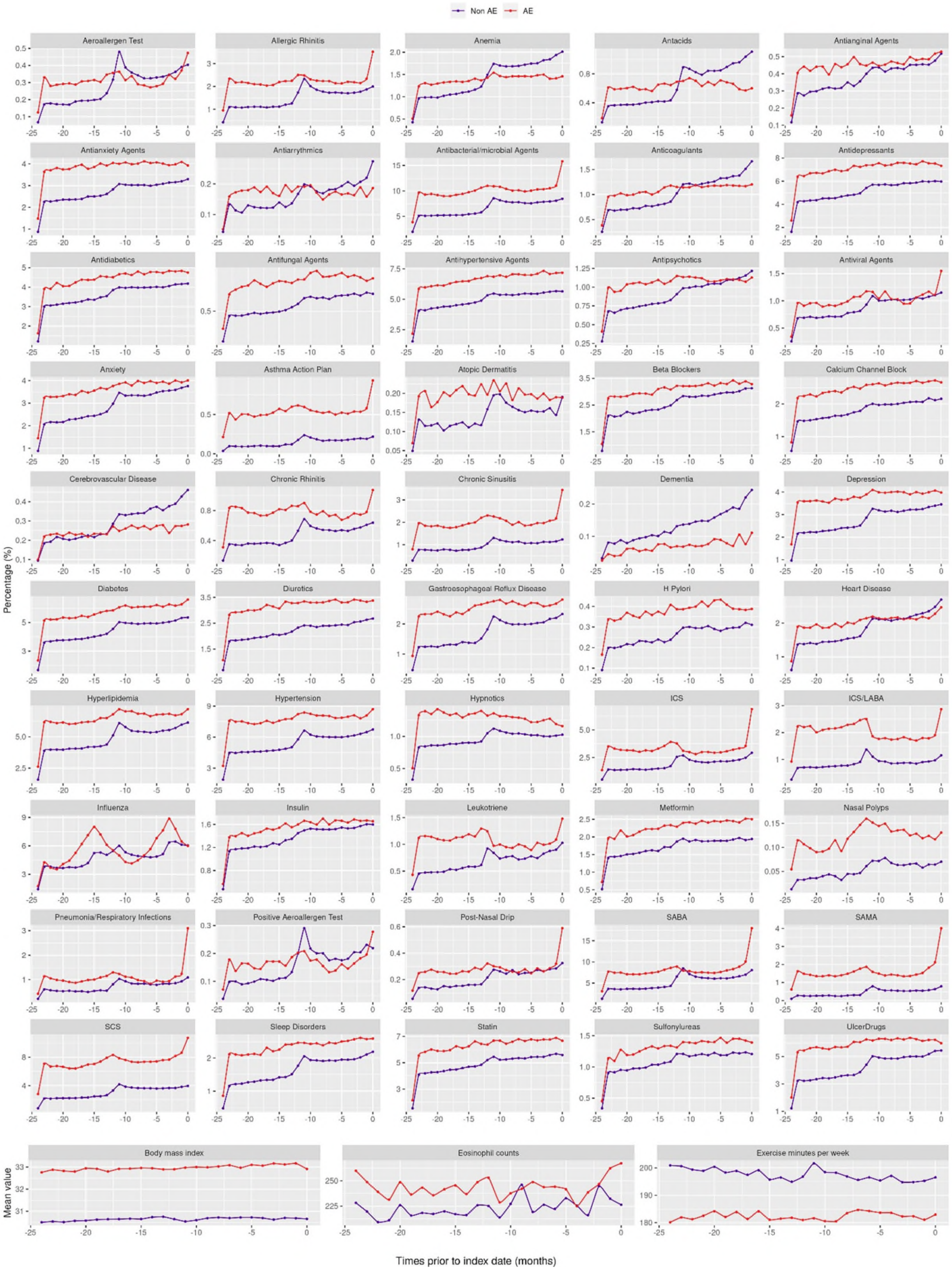

Supplementary Figure 5. Feature importance based on single-feature model AUCs for case–control dataset**.** Each bar shows the predictive performance (area under the ROC curve, AUC) of a model trained using only the indicated feature from the KPSC case–control dataset. Bars are color-coded by feature domain. Top-ranked features included systemic corticosteroids (SCS), body mass index (BMI), short-acting beta-agonist (SABA), antibacterial/antimicrobial agents, and exercise minutes per week.

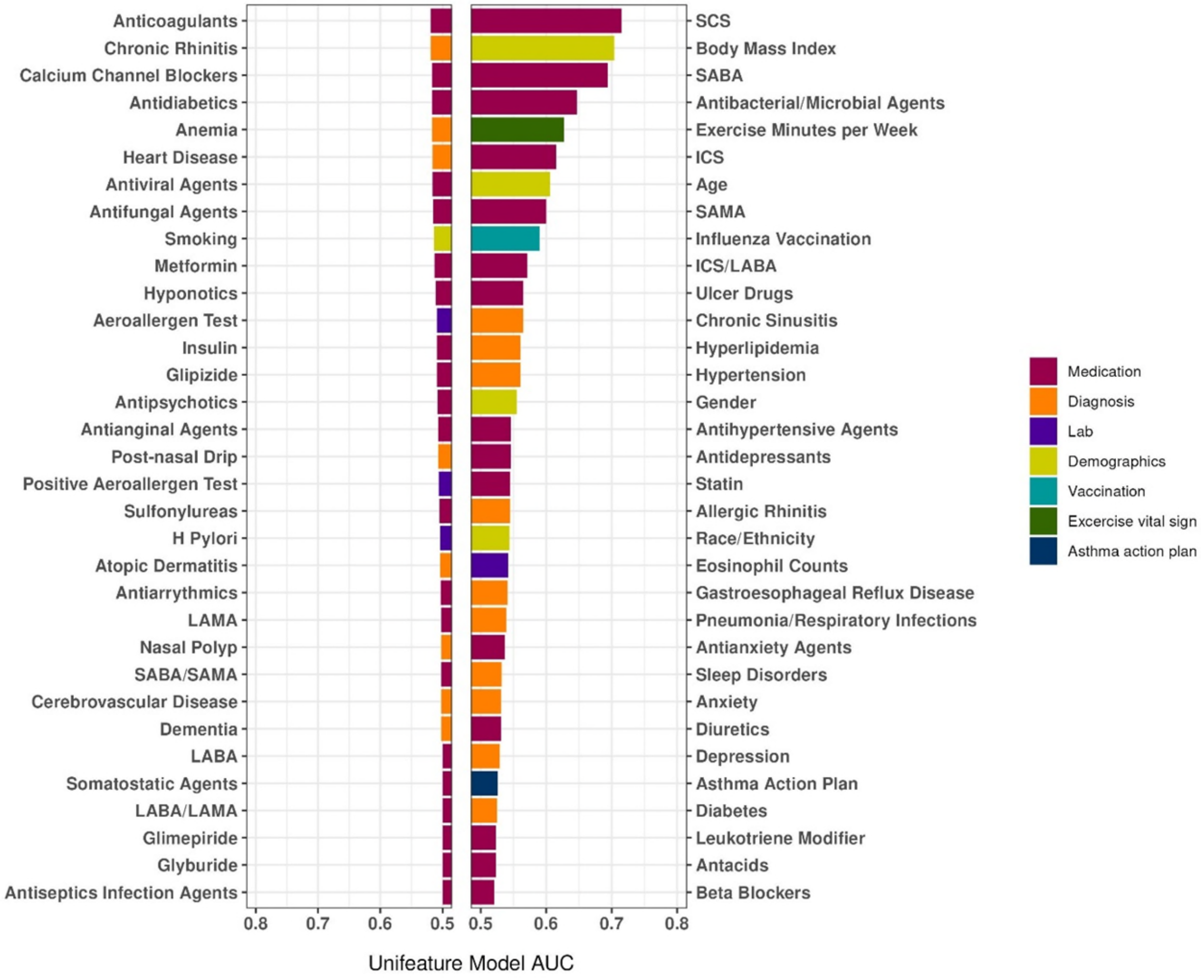

Supplementary Figure 6. Precision–recall–F1 performance across risk thresholds for the KPSC datasets. Precision (positive predictive value), recall (sensitivity), and F1 score plotted across a range of prediction thresholds for (a) cohort-based models and (b) case–control-based models. As thresholds increase, precision rises and recall declines.

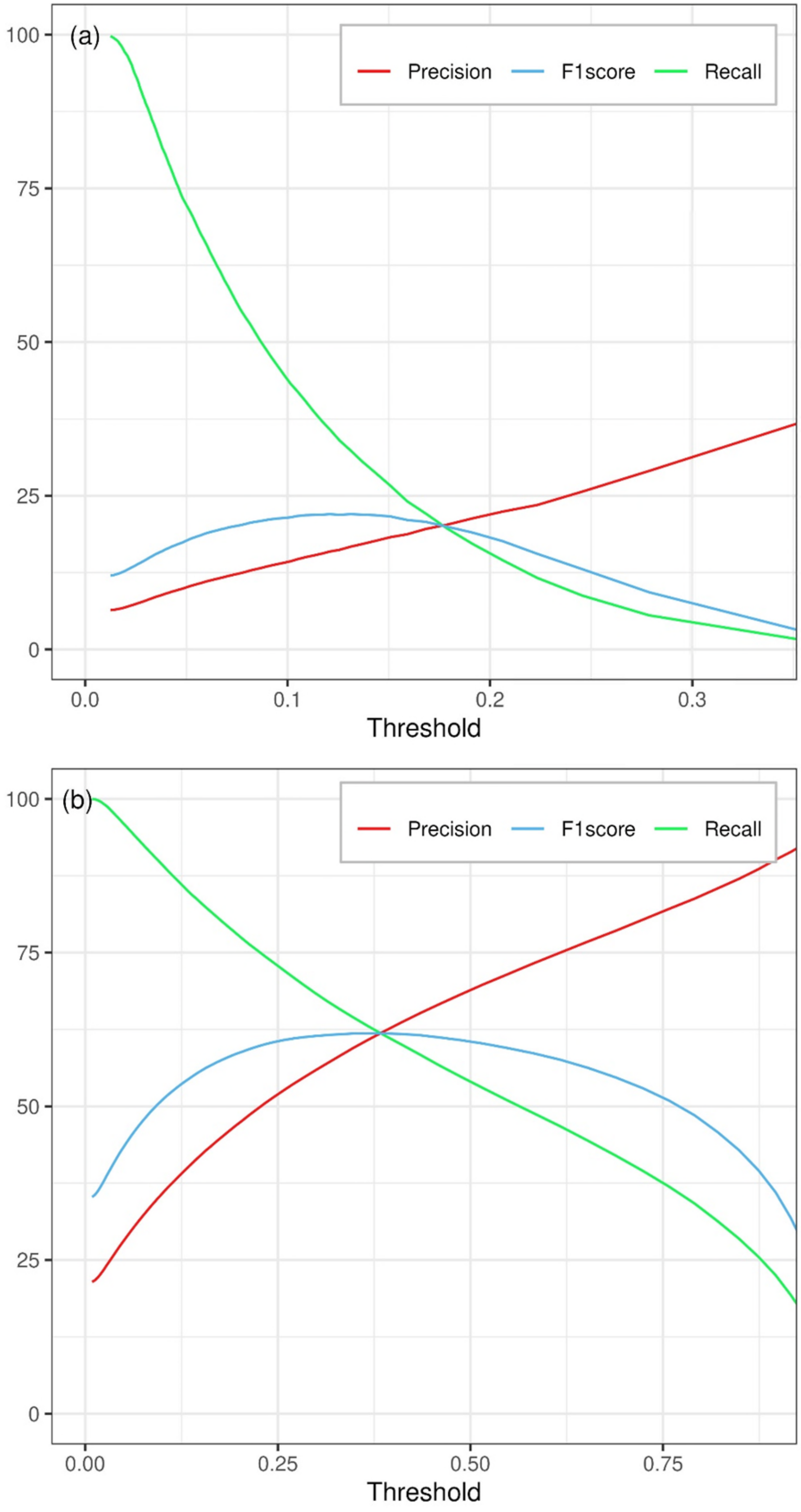

Supplement: Supplementary file 1 — PEARL risk prediction model development supplementary file R1 [file 41746_2026_2624_MOESM1_ESM.pdf]
